# Clinical microbiology laboratories and COVID-19: the calm before the storm

**DOI:** 10.2217/fmb-2020-0216

**Published:** 2020-11-06

**Authors:** Joseph M Blondeau

**Affiliations:** 1 Division of Clinical Microbiology, Royal University Hospital & Saskatchewan Health Authority; Departments of Microbiology & Immunology, Pathology & Laboratory Medicine, Ophthalmology, University of Saskatchewan, Saskatoon, Saskatchewan, Canada

**Keywords:** clinical microbiology, COVID-19, diagnosis

SARS-CoV-2 causing CoV disease or name assigned by the World Health Organization for SARS-CoV-2 is now reported from more than 213 countries and territories worldwide with devastating consequences reported from many countries in terms of the percentage of the population affected and deaths. In the 5 months since the pandemic was declared, more than 23 million people globally have been infected with more than 800,000 deaths and currently global epicenters include the USA, (∼5.9 million infected and ∼180,000 deaths), Brazil (∼3.6 million infected and ∼114,000 deaths), India (∼3.1 million infected with ∼58,000 deaths and Russia (∼960,000 infected and ∼16,000 deaths). Another 16 countries report >200,000 to >900,000 cases with total deaths per country ranging between 3300 and >55,000. These numbers are changing daily. In Canada, we have had approximately 124,000 cases with approximately 9000 deaths [1]. A report from Imperial College London (26 March 2020) estimated that in the absence of intervention, 7 billion infections and over 40 million deaths globally would have occurred [2]. A study published by Clark *et al.* estimated that 1.7 billion people, comprising 22% of the global population have at least one underlying health condition (i.e., chronic kidney disease, diabetes, cardiovascular disease and chronic respiratory disease) that puts them at an increased risk of severe COVID-19 infection; an estimated 349 million people are at a high risk for severe COVID-19 infection and would require hospitalization [3]. Interestingly, 6% of males are estimated to be at high risk as compared with 3% of females. African countries with high HIV/AIDS prevalence and small island nations with high diabetes rates along with countries with older populations share increased risks. In Canada, an estimated 27% of the population has at least one pre-existing health condition putting them at increased risk for severe COVID-19 infection and some 1.8 million would require hospitalization. In addition to risk factors identified above, hypertension, obesity and immunosuppression also require consideration [4]. Demands for accurate and rapid laboratory testing for COVID-19 and the ability to differentiate it from other pathogens (causing similar symptoms) is essential now and moving forward.

A predominant focus in the media has rightly been on intervention measures such as social or physical distancing, hand hygiene, surface decontamination, coughing and sneezing etiquette and self-isolation and/or quarantine for patients positive for COVID-19 or deemed high risk, in other words, travel from endemic areas or contact with a confirmed positive case. More recently and continuing are the debates regarding the use of masks and/or face shields either voluntary or mandatory [5,6]. For patients at risk of infection due to potential exposure, a 14 day self-isolation or quarantine was generally recommended. Medically, considerable acute care cases and bed shortages, intensive case admissions and insufficient numbers of ventilators [7] and respiratory support were and remain a concern in every country due to continually escalating infection numbers and an anticipated ‘second wave’ of infections. Supplies of personal protective equipment remain an issue. Terminologies such as ‘surge’ have been used to refer to increases in cases over short periods of time. In addition, the daily global (and country specific) cases including deaths serve to remind the public of the extent of the pandemic and its impact on humanity.

Over the past 20 years there have been a number of other outbreaks, but the extent of the populations affected has been substantially less than the current COVID-19 pandemic. Historically, the most deadly pandemic was the ‘Spanish flu’ of 1918–1920. It was estimated that 500 million people worldwide (about 33% of the global population) were infected with some 20–50 million people dying [8,9]. Vaccines and/or drugs for therapy were not available, nor were antibiotics to treat secondary bacterial infections. Death rates were highest in children <5 years of age, those 20–40 years of age and those >65 years of age. Interestingly, public health measures such as isolation, quarantine, personal hygiene, disinfectants and limiting of social gatherings were promoted, similar to what is being promoted with COVID-19. There are many parallels between the Spanish flu pandemic and COVID-19, with a few exceptions. First, the death rate with COVID-19 is highest in elderly patients and those with comorbidities. Milder influenza pandemics occurred in 1957–1958 (‘Asian flu’ caused by [A/H2N2]) and again in 1968 (‘Hong Kong flu’ caused by [A/H3N2]) with each of these pandemics causing an estimated 1–4 million deaths globally. Second, the role of clinical microbiology for the laboratory-based diagnosis of influenza was essentially nil. Third, real time reporting was unavailable during the Spanish flu. Indeed, today, the ability to rapidly develop and implement a new highly sensitive and specific diagnostic test is amazing as is the ability to mass produce for implementation into diagnostic laboratories.

Several outbreaks have occurred since the 1960s [10]. In Uganda in 1967, 519 people were infected with Marburg virus of which 478 died (fatality rate of 81%). In 1976 Ebola virus infected over 33,000 people in the Democratic Republic of Congo with more than 14,000 people dying (fatality rate of 44%). Nipah virus in Malaysia in 1999 killed 265/496 infected people (fatality rate of 53%). In 2002 an outbreak of SARS, in China, had a fatality rate of 10% where 774 of 8098 infected people died. In 2003 in China, H5N1 bird flu infected 861 people of which 455 died (fatality rate of 53%). Middle Eastern respiratory syndrome was described in Saudi Arabia in 2012 and 858 of 2494 infected people died (fatality rate of 35%). In China in 2013 the H7N9 bird flu infected 1568 patients of which 616 died (fatality rate of 39%). Influenza A H1N1 swine flu (2009) originated in the USA and Mexico and was estimated to have infected 1 billion people with deaths ranging between 123,000–203,000 people (fatality rate below 0.01%). Seasonal influenza is estimated to infect approximately 1,000,000,000 people worldwide with deaths ranging between 290,000–650,000 and a fatality rate below 0.01%. In 2020, Ebola virus and Plague outbreaks have been reported from the Democratic Republic of the Congo, Middle Eastern respiratory syndrome-CoV from Saudi Arabia, Qatar and UAE, Yellow fever from French Guiana and Mayotte (France), Ethiopia, Togo, Uganda, Republic of South Sudan and Gabon, an influenza variant of A (H1N2) from Brazil, measles from Burundi, Central African Republic and Mexico, Dengue fever from Chile and the French Territories of the Americas (French Guiana, Guadeloupe, Martinique, Saint-Martin, Saint-Barthelemy), Dracunculiasis (Guinea worm disease) in Ethiopia, and Lassa fever from Nigeria.

Aside from deaths and capacity issues with morgues and body storage related to COVID-19, little attention has focused on diagnostic laboratory services. For sure, chemistry, hematology, transfusion medicine and anatomical pathology are none-the-less important during this pandemic, however, they are outside the scope of this commentary.

Simply put, clinical microbiology laboratories have been overwhelmed with the COVID-19 pandemic, but not just for CoV testing in labs performing such testing. In fact, in the absence of more dedicated resources, clinical microbiology laboratories may be ill-prepared currently and for the potential increase in cases expected over the coming months.

We are a University Medical Center and our clinical microbiology laboratory serves three acute care adult hospitals and most recently a new children’s hospital that serves as the provincial referral center for pediatrics. We also process specimens from outpatients seen by family physicians. Prior to the pandemic, our laboratory processed upwards of 1400 patient specimens per day in bacteriology, virology (molecular/serology based), mycology and parasitology. We have a full molecular diagnostic program including 16S ribosomal sequencing and overall our laboratory has advanced technology including MALDI–TOF. Specimen numbers for COVID-19 in our laboratory have ranged from 200 to 600 per day (14–43% increase in daily specimen volumes) and current provincial plans will see us testing up to 1320 specimens/day at our site – a 94% increase in specimen numbers from pre-COVID-19 times. Our provincial plan is for testing 4000 COVID-19 specimens daily with two-thirds of these specimens being tested at the Provincial Public Health Laboratory. Our provincial population is approximately 1.2 million.

To deal with COVID-19 related testing, we enacted emergency measure services which essentially meant a reduction in community-based testing and focusing on emergency room, in-hospital acute case testing and critical care testing from the intensive care unit. Additional considerations for specimen testing included outbreak investigations (including institutionalized individuals) related to public health and long-term care residents.

Our site is one of the two main testing laboratories for COVID-19 in our province. We also have distributed limited volume testing in rural facilities with GeneXpert technology. With the anticipated increase in specimens for COVID-19 testing, laboratories faced several immediate challenges – some of which continue today and will for the foreseeable future. Our challenges can be broadly summarized in four major areas and are likely the same in all other laboratories doing COVID-19 testing: staffing, technology, training and supply chains. We used quality and process improvement specialists to help with our preparedness plan.

Regarding staffing we estimated a need for approximately ten additional laboratory personnel to perform testing specifically for COVID-19. In addition to hiring such people, we have developed a rigorous training program that would allow each new hire to be able to perform at least part of the test so that we can utilize these extra resources to assist with our daily testing demands. New staff have been approved from the Ministry of Health for a period up to two years to assist specifically with testing for COVID-19. These additional staff members will allow us to increase the number of shifts daily for COVID-19 testing and as such the number of specimens we can process daily. Part of our provincial strategy is for accurate and timely (turn-around time of <24 h for non-STAT [with no delay] related requests) reporting of results which in turn can be used by public health to advise patients on their need for self-isolation, quarantine and cluster of cases control.

Technology is a critical component of the preparedness plan. To this point we have expanded the number of nucleic acid extractors (both high and low volume, i.e., 24 vs 96 specimen capacity) as well as the number of Thermo cyclers. Due to the issues with supply chain (discussed below), we are operating nucleic acid extractors from three different manufacturers so as to ensure we maintain the ability to continue to offer testing daily – 7 days a week. We have also had to increase technology capacity, in other words, blood culture capacity in order to protect patients during surge capacity concerns (discussed later). Prior to the COVID-19 pandemic, our blood culture capacity was running at approximately 75%. We had determined that the 25% of available space for blood culture bottles was insufficient. Additionally, we introduced additional platforms for multiplex PCR for some specimens (i.e., stools for bacteria, viruses and parasites) in order to improve sensitivity and decrease technologist time in processing and interpreting specimens by culture and microscopy. Stools positive by PCR would subsequently be cultured to isolate bacterial pathogens for susceptibility testing.

At the start of the COVID-19 pandemic, supply chain was a nightmare. Numerous manufacturers found themselves in situations where demand greatly exceeded supply and as a consequence, many laboratories struggled to continue to offer testing in some areas. Certainly, manufacturers have responded by increasing production capacity and many laboratories including our own are now stockpiling supplies in anticipation of increased demand in the coming months. For some products, manufacturers have resorted to supply allocation (i.e., Genexpert) with a defined number of product being sent to labs weekly or monthly. Labs in such situations have had to rationalize these products in order to guarantee availability when needed. As new assays are being developed for COVID-19 and other respiratory viruses (i.e., BD Max) we have already been informed that assays will be allocated based on the defined number per site, such that each site using that assay would have access to at least a limited number. Having limitations on supplies clearly influence the ability to use that technology when higher assay demands are needed.

Training has been a critical component of our COVID-19 preparedness plan. Our microbiology laboratory is designed based on a ‘hub and spoke’ model whereby each of our medical laboratory technologists (MLTs) are trained and proficient in the bacteriology section and of these MLTs, a number are crossed trained in mycology or parasitology or our molecular section. This model works well for us as it allows us to have the staff necessary for days, evenings and weekend coverage and for STAT-related requests (i.e., required testing for the transplant service). When COVID-19 was declared a pandemic, we enacted an emergency services test menu (as mentioned) which allowed us to free up MLTs for cross-training in molecular on all the technology and protocols necessary for COVID-19 based on nucleic acid amplification testing. We increased the number of trained MLTs from nine to 23 (fully trained or partially trained for, i.e., extraction or amplification) and continue to recognize the need for additional staff to be trained.

Considerable focus has emphasized ‘COVID-19’ testing. In reality, testing for acute and critical care patients is essential during the pandemic. For example ‘order sets’ for acutely ill patients being admitted to our hospitals includes blood culture sets and respiratory specimens for culture and susceptibility – in addition to COVID-19 testing. Some patients may also require extended multiplex PCR testing for other respiratory viruses, as respiratory viruses other than COVID-19 have been simultaneously circulating during the pandemic. COVID-19 and other respiratory viruses yield similar symptoms – particularly in those with mild symptoms. Such an approach is not surprising as ordering of blood cultures (pre-COVID-19) is common. One study indicated that approximately 16–17% of blood cultures come from the emergency services and 35–40% of internal medicine admissions get blood cultures [11,12] and between 30 and 39% of patients get repeat blood cultures. Another study reported that 30.7, 13.9, 12.2 and 7.3% of blood cultures were ordered from the emergency department, hematology, intensive care unit and acute medical units, respectively [13].

Blood cultures remain among the most important and critical specimens processed by clinical microbiology laboratories. Between 30 and 50% of patients with suspected sepsis or shock have positive blood cultures [14] and blood stream infections are associated with 14–37% mortality [15,16]. Schwarzenbacher *et al.* recently commented that only 20% of laboratories in Europe offer 24 h microbiology services, thereby adding additional challenges for the timely reporting of results (COVID-19 or other pathogens) [17]. Idelevich *et al.* reported only 13% of laboratories from 25 European countries provide 24 h microbiology service [18]. Interestingly, in the report by Schwarzenbacher *et al.*, on-site incubation of blood cultures was associated with reduced time to knowledge of positivity (p < 0.001) and reduced time to result (p < 0.001) and the authors argued the importance of 24/7 diagnostics providing round the clock processing of blood culture specimens. Arguably the same can be made for other specimen types including those for COVID-19.

Langford *et al.* performed a systematic review of bacterial co-infection and secondary infection in patients with COVID-19 [19]. A total of 24 studies representing 3338 patients with COVID-19 were evaluated for acute bacterial infection. Bacterial co-infection was identified in 3.5% of patients and secondary bacterial infection in 14.3% of patients, with the overall proportion of COVID-19 patients with bacterial infection being 6.9%. In patients that were critically ill, bacterial infection was more common at 8.1%. The majority of patients with COVID-19 received antibiotics. In a letter to the editor, Zhou *et al.* indicated that bacterial and fungal infections in COVID-19 patients were a matter of concern [20]. From data summarized from three studies, secondary infection was found in 13–50% of COVID-19 nonsurviving patients as compared with 0–25% of patients who survived. Antibiotic use was similar in both surviving and nonsurviving patients and ranged from 93 to 100%. Regardless of the absolute percentage of acute or secondary bacterial infections, specimens from COVID-19 patients will continue to be submitted to microbiology laboratory for processing and analysis for pathogens other than COVID-19.

Restricting testing, while necessary, is not without risk. For example, bacteremia is frequently a common complication of urinary tract infection [21]. Complications of streptococcal pharyngitis may arise from direct extension of pharyngeal infection or by hematogenous and/or lymphatic spread [22]. Clearly, restricted test menus need to consider risks to patients.

Other pressures for testing are linked to international drug shortages [23]. For example, shortages of intravenous acyclovir [24] prompted neurologists and infectious diseases physicians to request PCR testing of cerebral spinal fluid as a necessary indicator for continuing or stopping therapy in patients empirically treated. In fact, testing to stop therapy was often ordered as ‘STAT’.

COVID-19 has served to remind us of several important things. First, outbreaks of infectious and transmissible pathogens occur more frequently than we give thought to and fortunately many of these are quickly contained or are geographically restricted. Second, international travel and globalization are components of the ‘perfect storm’ for the global dissemination of emerging pathogens. Third, limitations of supply chain when a sharp (sudden?) increase in demand for products or substituents of products needs to be addressed going forward and stockpiling is not the best or only solution. Fourth, clinical microbiology laboratories need to be opened 24/7 to provide STAT and critical care testing for optimal patient care. A robust and well-funded public health system, including microbiology laboratory support, is critical to be able to respond to emerging infectious diseases challenges. One report suggests COVID-19 could cost the global economy approximately $22 trillion in 2020 alone [25]. Arguably, spending a fraction of this on preparedness (labs, technology and personnel) and public health will make us better prepared for the next pandemic. Fifth, national and international collaboration on common goals requiring urgent resolution is essential for evidenced-based development of necessary interventions (i.e., diagnostic tests, drugs or vaccines). Sixth, public messaging campaigns delivering timely, factual and transparent information need to be both developed and repeatedly delivered as the public has a huge role to play in any pandemic. Never before in history did we have the means as we do today to use multiple different media platforms (including social media) to deliver messaging. While ‘COVID fatigue’ is a real phenomenon, it does not negate the important role the public has in the ongoing COVID-19 pandemic and /or new infectious disease entities that may arise. Seven, every problem also presents an opportunity and COVID-19 is a platform for reminding the public on the importance of vaccinations, including the annual influenza vaccine where vaccination rates vary considerably globally. This is another important arena for public messaging.

Are we in the ‘Calm before the Storm’? As we transition from summer to autumn and then to winter, we are likely to continue to see a rise in COVID-19 cases. The arrival of seasonal respiratory viruses, including Influenza A/B and respiratory syncytial virus and the need to identify the infecting pathogen will further strain laboratories. The transmission of COVID-19 from asymptomatic individuals coinciding with relaxation of social restriction argues for broader testing [26]. Leung *et al.* commented on the potential consequences of premature relaxation of lockdown in the absence of safe and effective vaccines [27]. Such an increase in cases could result in higher health and economic losses. A decision to relax restrictions should be tailored to local situations and linked to increased testing [28]. Collectively, clinical microbiology laboratories performing COVID-19 will face tremendous pressures in the coming months. In our institution, the resilience of our team has kept us afloat this far and we continue to rely on our dedication and ingenuity to see us through the coming months. STAT COVID-19 testing requests on inpatients (including patients in the emergency room) may become a more frequent request 24 h a day and as always, clinical microbiology laboratories will rightly need to adjust.
